# Infectious disease risk in asbestos abatement workers

**DOI:** 10.1186/1471-2458-12-665

**Published:** 2012-08-16

**Authors:** John H Lange, Giuseppe Mastrangelo, Luca Cegolon

**Affiliations:** 1 Envirosafe Training and Consultants, Pittsburgh, PA, USA; 2 Padua University, Department of Molecular Medicine, Padua, Italy; 3 Imperial College London, School of Public Health, St. Mary's Campus, London, UK

## Abstract

**Background:**

The current literature reports increased infectious disease occurrence in various construction occupations, as an important contributor to morbidity and mortality arising from employment.

These observations should be expanded to asbestos abatement workers, as the abatement can create an environment favorable for bacterial, viral and fungal infections.

**Discussion:**

Asbestos abatement work employs activities resulting in cuts, blisters and abrasions to the skin, work in a dirty environment and exposure to dust, mists and fumes.

Furthermore, this population exhibits a high smoking rate which increases the risk of chronic obstructive pulmonary disease and respiratory infections.

In addition, these workers also commonly employ respirators, which can accumulate dirt and debris magnifying exposure to microbes. Use of respirators and related types of personal protective equipment, especially if shared and in the close environment experienced by workers, may enhance communicability of these agents, including viruses.

**Summary:**

Abatement workers need to be provided with information on hazards and targeted by appropriate health education to reduce the infection risk. Epidemiological studies to investigate this risk in asbestos removers are recommended.

## Background

Several studies [1-5] have reported on increased infectious disease occurrence in various construction occupations, with these agents constituting an important contributor to morbidity and mortality arising from employment.

We believe these observations need to be expanded to asbestos abatement workers, especially since infectious diseases appear to be associated with certain industrial occupations, most notably those in the construction industry.

Respiratory diseases are commonly discussed as a hazard for those employed in the asbestos abatement industry, although focus is on cancer and asbestosis. Recently there has even been a suggestion of exposure to asbestos contributing to cardiovascular disease [6] with this agent causing disease states in locations other than the lungs and those traditionally identified, especially at high exposure levels [7]. For this group of workers there are periodically discussions of other non-asbestos hazards (e.g. confined conditions, heat disorders, etc.), with the literature rarely mentioning infectious diseases [8,9].

## Discussion

One study [1] reported exposure to inorganic dust, which includes asbestos, for construction workers resulted in a high rate of mortality from infectious pneumonia (relative risk for inorganic dust =1.87, with 95% confidence interval: 1.22-2.89). Since asbestos abatement workers are exposed to both inorganic/mineral dusts and chemicals, it would appear a risk from infectious disease exists and include various forms of infections (e.g. skin) beside mortality from pneumonia as indicated by Thoren [1] for inorganic dust.

A PubMed search of “asbestos abatement and infectious disease”, “asbestos abatement and infections”, “asbestos workers and infectious disease” or “asbestos workers and infections” produced no actual citations; although, one existed as related to fungal exposure during abatement [10]. Today, mold (fungal) exposure during abatement is recognized as a hazard for this type of industry [11,12].

Overall, abatement can create an environment favorable for bacterial and fungal infections [13]. For construction workers, most reports discuss infections related to bacterial or fungal diseases [3,13,14], but have not clearly illustrated how this hazard extends to asbestos-related work. This report may be the first in the open literature expressing the concept of infectious disease risk directly linked to asbestos abatement workers.

Asbestos abatement work employs activities resulting in cuts, blisters and abrasions to the skin, work in a dirty environment and exposure to dust, mists and fumes (i.e. inorganic and mineral dusts). Exposures and operations of this nature place this group (abatement workers in general) at risk for skin and mucous membrane, uro-genital tract, respiratory, gastro-intestinal tract, cutaneous, direct contact, animal reservoirs-related and inanimate grouped infections as classified by Haagsma [2]. Infection risk in construction as well as in asbestos abatement work should probably be considered similar to other industries’ that a lay person may consider riskier in terms of communicable diseases (e.g. underground mining [15-17], agriculture [18,19], cattle farming [20], sewerage work [21-24], infantry in military [25], etc.).

Furthermore, asbestos abatement workers exhibit a high smoking rate (e.g. 70%) [26], which increases the risk of chronic obstructive pulmonary disease (COPD) and respiratory infections. It has also been observed that even non-smokers exposed to inorganic dust have an increased mortality rate for COPD [27], enhancing the potential of infection.

In addition, these workers also commonly employ respirators, which can accumulate dirt and debris magnifying exposure to microbes. Use of respirators and related types of personal protective equipment, especially if shared and in the close environment experienced by workers, may enhance communicability of these agents, including viruses. Moreover dermatitis from various causes (e.g. chemical and infectious agents) is a concern [28].

With these findings, type of work performed by abatement workers and their high rate of smoking, it appears this occupational group is at increased risk for various types of infectious disease, including those associated with the pulmonary system.

This hazard may be of even greater importance for asbestos abatement workers in terms of health consequences (e.g. emerging as an important risk factor) since current exposure to asbestos has been shown to be low and there is now little risk of asbestos-related disease for this population (asbestos abatement workers – in the western world) [29,30].

When discussing non-asbestos disease hazards for this occupational population, infectious (microbial) agents should be included along with hygiene practices related to prevention. Abatement workers need to be provided with information on hazards and prevention of infectious disease and dermatitis; the Internet can be a very helpful resource in this complex and changing field if used efficiently [31]. Personnel also need to be targeted by health education aimed at reducing the infection risk [32]. Decontamination and hand washing would appear to be of great importance, but is not the only form of protection/prevention available (e.g. disinfection agents), especially for dermatological and gastro-intestinal risks/hazards. Abatement workers have routine physical examinations primarily focusing on the pulmonary system and the ability to use a respirator. However, in many cases this has become a bureaucratic paper process acting as a work permit and not functioning as a true evaluation of health [33]. Regardless, it may be prudent during this evaluation also to check these workers for their current level of vaccination (e.g. against Pneumococcus, Haemophilus influenza type b and tetanus toxoid), and according to the type and location of work other vaccines could be considered, where appropriate and feasible [34]. In many ways, this is a forgotten aspect of occupational medicine, especially for those undertaking various forms of abatement. A major role can be played by general practitioners and occupational physicians in terms of health education. These preventive measures can be applied to the construction-related industries in general.

## Summary

The underestimation of the infectious risk among asbestos removers is likely due to the limited number of epidemiological studies on asbestos abatement workers as compared to asbestos workers.

Indeed, as they are exposed to both inorganic/mineral dusts and chemicals, these workers would appear at risk from various forms of infections and communicable diseases. Therefore an evaluation of this population regarding infectious disease occurrence seems to be recommended to determine the extent of this hazard as suggested more broadly by Haagsma [2]. Lastly, abatement workers need to be provided with information on hazards and targeted by appropriate health education to reduce the infection risk.

## Competing interests

The authors declare that they have no competing interests.

## Authors’ contributions

JHL conceived the idea and drafted the paper; GM contributed to the drafting of the paper; LC drafted the paper and had general responsibility of the work. All authors read and approved the final manuscript.

## Pre-publication history

The pre-publication history for this paper can be accessed here:

http://www.biomedcentral.com/1471-2458/12/665/prepub
